# Deciphering complex antibiotic resistance patterns in *Helicobacter pylori* through whole genome sequencing and machine learning

**DOI:** 10.3389/fcimb.2023.1306368

**Published:** 2024-01-04

**Authors:** Jianwei Yu, Yan Jia, Qichao Yu, Lan Lin, Chao Li, Bowang Chen, Pingyu Zhong, Xueqing Lin, Huilan Li, Yinping Sun, Xuejing Zhong, Yuqi He, Xiaoyun Huang, Shuangming Lin, Yuanming Pan

**Affiliations:** ^1^ Department of Gastroenterology, Longyan First Affiliated Hospital of Fujian Medical University, Longyan, Fujian, China; ^2^ Department of Gastroenterology, the 7Medical Center of PLA General Hospital, Beijing, China; ^3^ Center for Systems Biology, Intelliphecy, Main Building, Beishan Industrial Zone, Shenzhen, Guangdong, China; ^4^ College of Life Sciences, University of Chinese Academy of Sciences, Beijing, China; ^5^ Department of Gastroenterology, Xiamen Humanity Hospital, Xiamen, Fujian, China; ^6^ Department of Gastroenterology, Peking University Aerospace School of Clinical Medicine, Beijing, China; ^7^ Department of Data Science, Intelliphecy, Nanjing, Jiangsu, China; ^8^ Department of Nephrology, Longyan First Affiliated Hospital of Fujian Medical University, Longyan, Fujian, China; ^9^ Department of Science and Education, Longyan First Affiliated Hospital of Fujian Medical University, Longyan, Fujian, China; ^10^ Department of Gastroenterology, Beijing Chest Hospital, Capital Medical University, Beijing Tuberculosis and Thoracic Tumor Research Institute, Beijing, China; ^11^ Department of Gastrointestinal Surgery, Longyan First Affiliated Hospital of Fujian Medical University, Longyan, Fujian, China; ^12^ Cancer Research Center, Beijing Chest Hospital, Capital Medical University, Beijing Tuberculosis and Thoracic Tumor Research Institute, Beijing, China

**Keywords:** antimicrobial resistance (AMR), Helicobactor pylori, genomic sequencing data, machine learning methods, molecular mechanism

## Abstract

**Introduction:**

Helicobacter pylori (H.pylori, Hp) affects billions of people worldwide. However, the emerging resistance of Hp to antibiotics challenges the effectiveness of current treatments. Investigating the genotype-phenotype connection for Hp using next-generation sequencing could enhance our understanding of this resistance.

**Methods:**

In this study, we analyzed 52 Hp strains collected from various hospitals. The susceptibility of these strains to five antibiotics was assessed using the agar dilution assay. Whole-genome sequencing was then performed to screen the antimicrobial resistance (AMR) genotypes of these Hp strains. To model the relationship between drug resistance and genotype, we employed univariate statistical tests, unsupervised machine learning, and supervised machine learning techniques, including the development of support vector machine models.

**Results:**

Our models for predicting Amoxicillin resistance demonstrated 66% sensitivity and 100% specificity, while those for Clarithromycin resistance showed 100% sensitivity and 100% specificity. These results outperformed the known resistance sites for Amoxicillin (A1834G) and Clarithromycin (A2147), which had sensitivities of 22.2% and 87%, and specificities of 100% and 96%, respectively.

**Discussion:**

Our study demonstrates that predictive modeling using supervised learning algorithms with feature selection can yield diagnostic models with higher predictive power compared to models relying on single single-nucleotide polymorphism (SNP) sites. This approach significantly contributes to enhancing the precision and effectiveness of antibiotic treatment strategies for Hp infections. The application of whole-genome sequencing for Hp presents a promising pathway for advancing personalized medicine in this context.

## Introduction

*Hp* infection as the gram-negative pathogen, was estimated to infect over 50% of the world population, which is the cause for chronic gastritis (Salih, 2009). There are more evidences of the adverse events of *Hp* infection, patients with chronic *Hp* infection were more likely to have gastric cancer in their lifetime (Wroblewski et al., 2010), and Combination of antibiotics and mucosa protection drug is the standard of care for *Hp* infected patients. However, antibiotic resistance posed a significant challenge in clinical practice. The resistance of *Hp* to amoxicillin and tetracycline was as high as 9% and 15% respectively in a north China cohort (Wang et al., 2019). Both amoxicillin and tetracycline were first-line antibiotics used in clinic. Trial and error strategy not only increased cost and treatment time for the patients, but also renders the patients a higher probability of side effects. Antimicrobial susceptibility testing (AST) was adopted in clinic for patients who have failed standard treatment options (Smith et al., 2014). However, AST was limited by the culturing method and typically required a lengthy time.

Next generation sequencing (NGS) has emerged to be a powerful technology to characterize microbiome in the past decade (Malla et al., 2018). NGS allows the analysis of microbial composition within a sample and microbial subtyping with in-depth sequencing and SNP calling. NGS derived bacterial genotype has been related to phenotypes under a variety of circumstances (van Opijnen and Camilli, 2012; Dutilh et al., 2013; Mousavizadeh and Ghasemi, 2021). It has been reported that mutations in 23S rRNA were associated with clarithromycin resistance in *Hp* (Versalovic et al., 1996). Previous studies have shown that phenotypic resistance correlates well with genetic tests, while failed to provide high confidence single locus to accurately describe resistant patterns for different antibiotics (Camorlinga-Ponce et al., 2020). The inaccuracy of predicting antibiotic resistance was partially attributable to incomplete penetration of mutations in dictating phenotypes. Researchers explored the feasibility to use genotype-based machine learning methods to model the antimicrobial resistance of Actinobacillus pleuropneumoniae from Whole genome sequencing (WGS) data (Liu et al., 2020). Currently, systemic effort to address the antibiotic resistance of *Hp* using supervised machine learning is still lacking.

In this study, we established a workflow measure the phenotype and genotype of Hp strains, including Hp culturing and single colony picking, disk diffusion test and whole genome sequencing. We interrogated the antibiotic resistance of *Hp* by combining whole genome sequencing and machine learning approach. SNP was determined for Hp strains and used as input for modeling antibiotic resistance, with single SNP modeling, unsupervised and supervised machine learning. The goal is to explore the possibility to use WGS in clinical setting to predict antimicrobial resistance.

## Results

### Summary of patient cohort

A comprehensive summary of the patient cohort enrolled in our study is presented in **Supplementary Table 1**. With patient age spanning from 23 to 71 years, the broad age distribution underscores the extensive impact of *Hp* infection across the entire population. The diverse age range vividly reflects the ubiquitous nature of *Hp*’s influence. Notably, *Hp* isolates derived from these patients underwent rigorous drug testing to ascertain their susceptibility to six antibiotics, namely Amoxicillin, Clarithromycin, Levofloxacin, Metronidazole, Tetracycline, and Furazolidone. Interestingly, 86.5% of strains (45/52) are resistant to Metronidazole.

The statistic for enrolled patients and analyzed strains are summarized in **Supplementary Table 2**. The patient cohort included 21/52 (40.4%) male patients and 31/52 (59.6%) female patients. The median age is 50. The rate of resistance are 17.3% for Amoxicillin, 44.2% for Clarithromycin, 38.5% for Levofloxacin and 86.5% for Metronidazole. All strains are sensitive to Furazolidone. Only one strain is resistant to Tetracycline.

**Figure 1:**
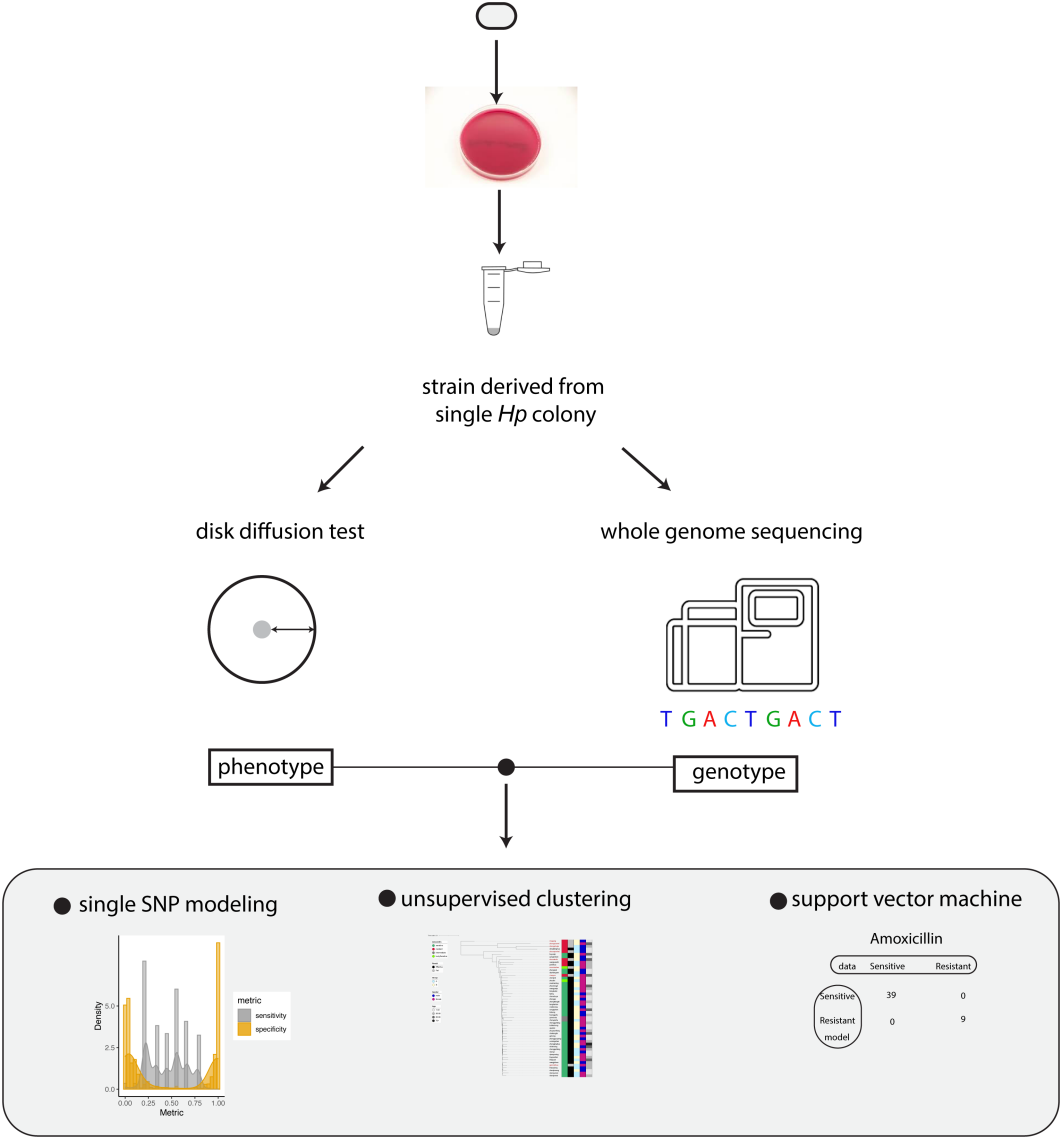
Summary of the workflow. Schematic summary of our study. The phenotype and genotype of *Hp* strains were quantitatively measured and the relationship between *Hp* phenotype and genotype was modeled by univariate analysis, unsupervised clustering and support vector machine.

To investigate how the genotype information of *Hp* can be used to predict antibiotic resistance, we established a workflow to measure drug sensitivity phenotype using disk diffusion test and genotype using whole genome sequencing (**Figure 1**). The relation between genotype and phenotype was modeled using univariate statistical test, unsupervised machine learning (phylogenetic analysis) and supervised machine learning (support vector machine).

### Overview of SNPs

The pivotal aspect of our investigation involved subjecting *Hp* isolates obtained from the patients to cutting-edge next-generation sequencing. This technological approach facilitated the comprehensive acquisition of whole genome data. To ensure the reliability and quality of our findings, patients lacking accurate clinical data were thoughtfully excluded, as were patients yielding insufficient SNP data due to data quality concerns. Seven patients were excluded because lack of clinical information. Five patients were removed from downstream analysis as the total number of SNP is less than 3000. This stringent curation led to the inclusion of a robust cohort comprising 52 patients. In total, our study unveiled a median of 66,198 SNPs identified among these patients. The significant SNPs for Amoxicillin, Clarithromycin, Levofloxacin, Metronidazole and Tetracycline were summarized in (**Supplementary Tables 3–7**).

### Modeling approaches

Leveraging this pool of extracted SNPs, we conducted a series of rigorous downstream analyses. These analyses encompassed single SNP statistical tests, the construction of phylogenetic trees, and the implementation of supervised machine learning using Support Vector Machine. This multi-faceted approach allowed us to draw comprehensive insights into the genetic intricacies underpinning antibiotic resistance in *Hp* strains within our patient cohort.

Three modeling approaches were undertaken to address the relationship between antibiotic resistance and genotype, including univariate statistical test, unsupervised machine learning and supervised machine learning. Univariate statistical test serves as a foundational method to identify basic relationships and differences between resistant and sensitive strains. This approach is valuable for its simplicity and clarity, providing an initial understanding of the data. For the unsupervised machine learning component, phylogenetic analysis was employed. Phylogenetic trees facilitates the visualization and clustering of different strains, offering insights into how genetic variations might influence antibiotic resistance. For supervised machine learning, the Support Vector Machine (SVM) algorithm was selected for its effectiveness in classification tasks, especially in high-dimensional spaces. This approach is particularly beneficial when labeled data is available and predictive modeling is desired.

To evaluate the model performance, we used sensitivity and specificity throughout our study. Sensitivity and specificity are two key metrics used to evaluate the performance of a diagnostic test or model. Briefly, in our study sensitivity measures the proportion of actual positives (resistant strains) that are correctly identified by the model. Specificity measures the proportion of actual negatives (sensitive strains) that are correctly identified by the model.

### Prediction of amoxicillin resistance

**Figure 2:**
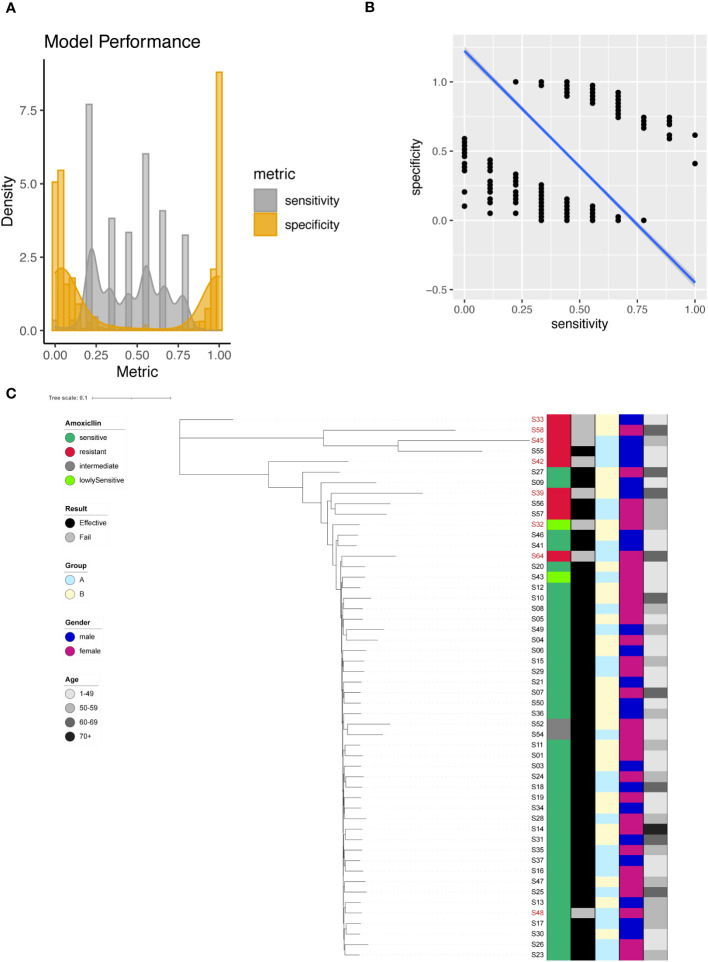
Amoxicillin resistance modeling using phylogenetic tree construction and single SNP classification. **(A)** Phylogenetic tree for Amoxicillin. Amoxicillin resistance is color coded indicating sensitive (Dark Green), resistant (Red), intermediate (Grey) and lowly sensitive (Light Green); Treatment result is color coded indicating Effective (Black) and Failed (Grey); Group is color coded to indicate two different gastric mucosa protection drugs A (Sky Blue) and B (Light Yellow); Gender is color coded to indicate Male (Blue) and Female (Red); Age is color coded to indicated different ranges, with darker color indicating larger age. **(B)** Sensitivity and specificity distribution for the models derived from single SNP site to predict Amoxicillin resistance. **(C)** Relationship between model sensitivity and model specificity was visualized as scatter plot. Blue line is fitted linear model.

We identified 2483 significant SNPs for Amoxicillin (**Supplementary Table 3**), including one significant site on 23S rRNA (A1834G). Using individual SNP for Amoxicillin resistance prediction, we evaluated the model with sensitivity and specificity. In this case the presence of a SNP suggested that the sequenced strain was an Amoxicillin resistant one. This assumption allowed us to determine the sensitivity and specificity of the individual models based on one single SNP site. We plotted the sensitivity and specificity of all resulted models (**Figure 2A**). The sensitivity ranged from 0 to 1, with a mean sensitivity of 0.45. The specificity also ranged from 0 to 1, with a mean specificity of 0.46. It was difficult to obtain a good tradeoff between model sensitivity and specificity (**Figure 2B**). For A1834G of 23S rRNA, the model achieved 100% specificity and only 22.2% sensitivity.

To enhance model performance, we took all 2483 significant sites as input for the construction of phylogenetic tree. The phylogenetic tree indicated that sensitive and resistant strains were well separated (**Figure 2C**).

### Prediction of clarithromycin resistance

**Figure 3:**
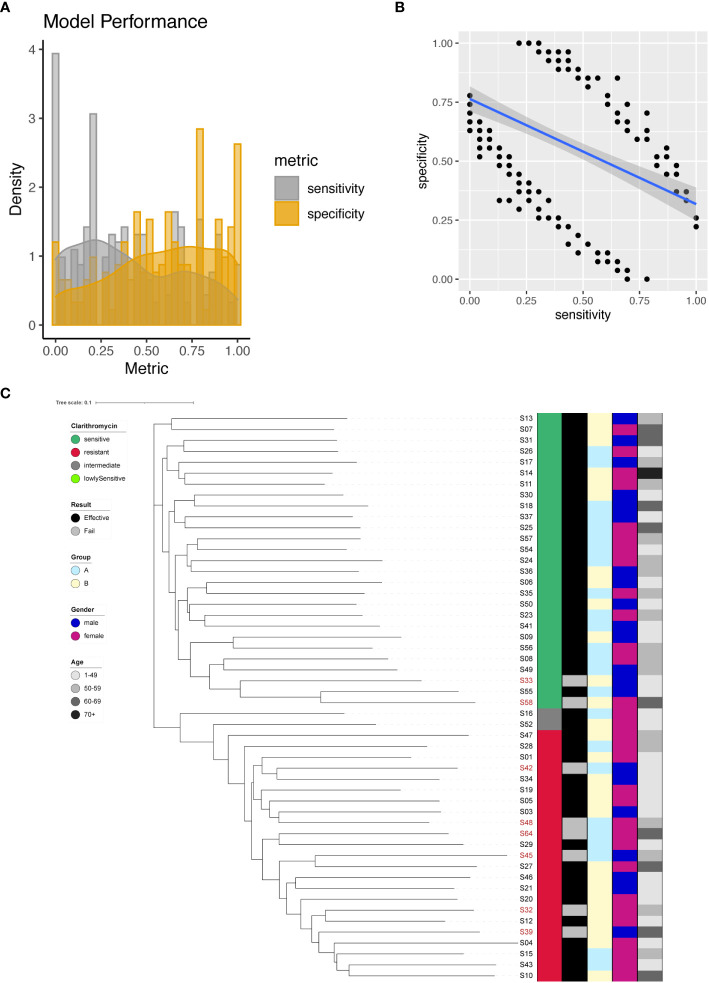
Clarithromycin resistance modeling using phylogenetic tree construction and single SNP classification. **(A)** Phylogenetic tree for Clarithromycin. Clarithromycin resistance is color coded indicating sensitive (Dark Green), resistant (Red), intermediate (Grey) and lowly sensitive (Light Green); Treatment result is color coded indicating Effective (Black) and Failed (Grey); Group is color coded to indicate two different gastric mucosa protection drugs A (Sky Blue) and B (Light Yellow); Gender is color coded to indicate Male (Blue) and Female (Red); Age is color coded to indicated different ranges, with darker color indicating larger age. **(B)** Sensitivity and specificity distribution for the models derived from single SNP site to predict Clarithromycin resistance. **(C)** Relationship between model sensitivity and model specificity was visualized as scatter plot. Blue line is fitted linear model.

In our investigation, we identified a total of 266 significant SNPs associated with Clarithromycin resistance (**Supplementary Table 4**), among which a noteworthy site was found on the 23S rRNA gene (A2147G). To predict Clarithromycin resistance, we utilized individual SNPs as predictive markers and assessed the model’s performance. The model’s effectiveness was evaluated by plotting the sensitivity and specificity of the resulting models (**Figure 3A**). Sensitivity values ranged from 0 to 1, with a mean sensitivity of 0.4, while specificity values ranged from 0 to 1, with a mean specificity of 0.58. Notably, except for A2147G, achieving a balanced tradeoff between model sensitivity and specificity proved to be challenging (**Figure 3B**). However, for the specific case of A2147G on the 23S rRNA gene, the model demonstrated 87% sensitivity and 96% specificity.

To further enhance the predictive capabilities of the models, we incorporated all 266 significant SNP sites as input for the construction of a phylogenetic tree. The outcome of this phylogenetic analysis indicated that strains with different sensitivities to Clarithromycin could be distinctly separated (**Figure 3C**). This result highlighted that through phylogenetic analysis, different strains of Helicobacter pylori were distinctly categorized based on their individual reactions to the antibiotic Clarithromycin, as evidenced by their separation in the phylogenetic tree.

### Prediction of Levofloxacin resistance

**Figure 4:**
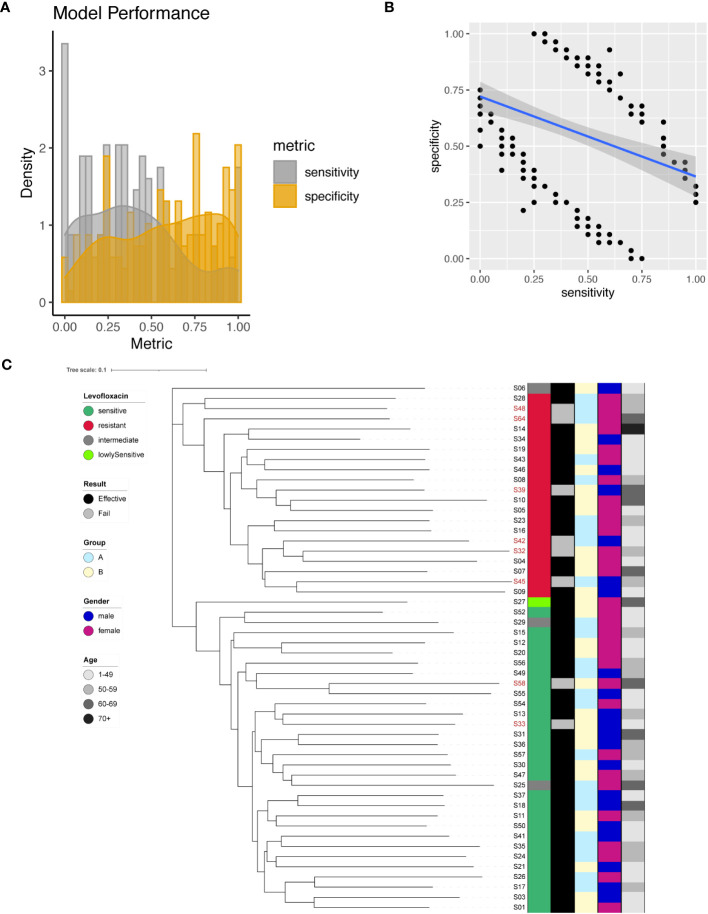
Levofloxacin resistance modeling using phylogenetic tree construction and single SNP classification. **(A)** Phylogenetic tree for Levofloxacin. Levofloxacin resistance is color coded indicating sensitive (Dark Green), resistant (Red), intermediate (Grey) and lowly sensitive (Light Green); Treatment result is color coded indicating Effective (Black) and Failed (Grey); Group is color coded to indicate two different gastric mucosa protection drugs A (Sky Blue) and B (Light Yellow); Gender is color coded to indicate Male (Blue) and Female (Red); Age is color coded to indicated different ranges, with darker color indicating larger age. **(B)** Sensitivity and specificity distribution for the models derived from single SNP site to predict Levofloxacin resistance. **(C)** Relationship between model sensitivity and model specificity was visualized as scatter plot. Blue line is fitted linear model.

We identified a total of 200 significant SNPs associated with Levofloxacin resistance (**Supplementary Table 5**), among which a significant site was found on the 23S rRNA gene (A2147G). To predict Levofloxacin resistance, we employed individual SNPs as predictive markers and evaluated the model performance. The performance metrics of the resultant models were assessed by plotting sensitivity and specificity (**Figure 4A**). Sensitivity values ranged from 0 to 1, with a mean of 0.4, while specificity values ranged from 0 to 1, with a mean of 0.58. Interestingly, there appeared to be a negative correlation between sensitivity and specificity (**Figure 4B**). Notably, the model for A2147G of the 23S rRNA gene achieved 65% sensitivity and 71% specificity.

To maximize the separation in resistant and sensitive strains, we utilized all 200 significant SNP sites as input to construct a phylogenetic tree. The resulting phylogenetic tree exhibited distinct separation between Levofloxacin-sensitive and resistant strains (**Figure 4C**). This phylogenetic analysis provided additional support for the differentiation between bacterial strains based on their Levofloxacin resistance profiles.

### Prediction of Metronidazole resistance

**Figure 5:**
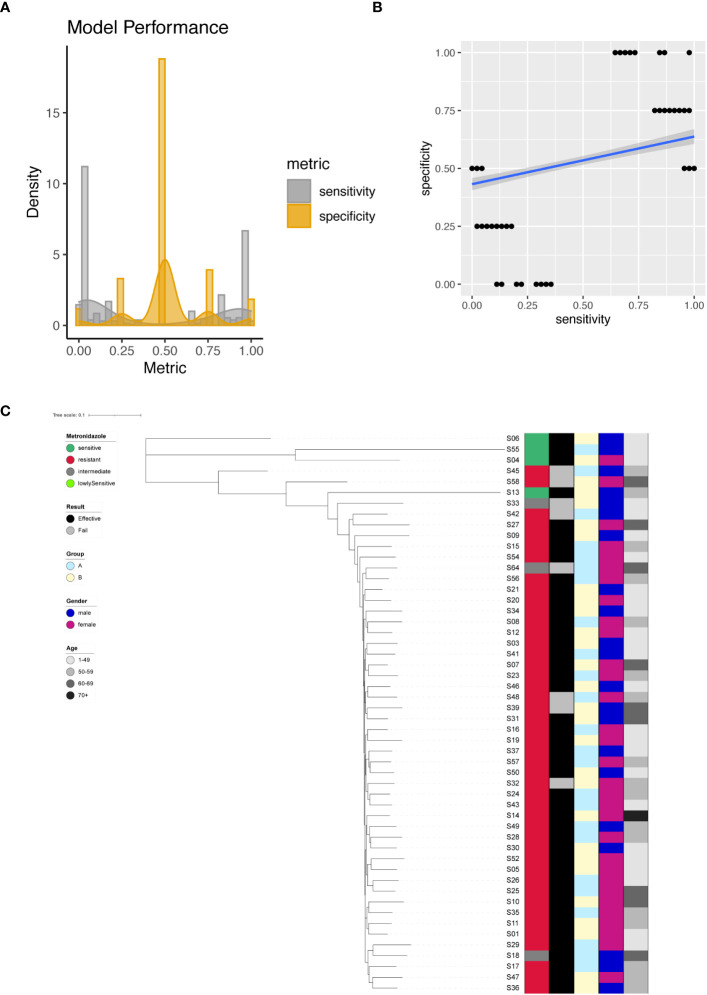
Metronidazole resistance modeling using phylogenetic tree construction and single SNP classification. **(A)** Phylogenetic tree for Metronidazole. Metronidazole resistance is color coded indicating sensitive (Dark Green), resistant (Red), intermediate (Grey) and lowly sensitive (Light Green); Treatment result is color coded indicating Effective (Black) and Failed (Grey); Group is color coded to indicate two different gastric mucosa protection drugs A (Sky Blue) and B (Light Yellow); Gender is color coded to indicate Male (Blue) and Female (Red); Age is color coded to indicated different ranges, with darker color indicating larger age. **(B)** Sensitivity and specificity distribution for the models derived from single SNP site to predict Metronidazole resistance. **(C)** Relationship between model sensitivity and model specificity was visualized as scatter plot. Blue line is fitted linear model.

We successfully identified a total of 379 significant SNPs associated with Metronidazole resistance (**Supplementary Table 6**), among which an important site on the 23S rRNA (G1517A) stood out. Employing individual SNPs for Metronidazole resistance prediction, we evaluated the performance metrics of our models. The sensitivity and specificity of all resulting models were plotted and analyzed (**Figure 5A**). The spectrum of sensitivity scores ranged between 0 and 1, with an average sensitivity value of 0.41. Correspondingly, specificity scores ranged between 0 and 1, yielding an average specificity of 0.52. Remarkably, one single SNP model emerged as an outlier, achieving nearly 100% sensitivity and an impressive 100% specificity (**Figure 5B**). However, when focusing on the known G1517A variant of the 23S rRNA, the model exhibited only 2% sensitivity and 50% specificity.

For unsupervised clustering of all strains based on SNP information, we leveraged all 379 significant sites as input for the construction of a phylogenetic tree. The resulting tree compellingly demonstrated that Metronidazole-sensitive and resistant strains could be distinctly separated (**Figure 5C**).

### Assessing clinical treatment outcomes

Furthermore, we conducted a comprehensive investigation into the potential impact of two different gastric mucosal protective agents (Group A and B) on treatment outcomes for *Hp* infections. More information about treatment scheme is documented in method. *Hp* is eradicated in 83.3% of strains treated with A and 85.7% of strains treated with B (*p* = 1). For the eight strains not successfully eradicated by Amoxicillin and Clarithromycin, four strains are resistant to both Amoxicillin and Clarithromycin and the other four stains are resistant to either Amoxicillin (n =2) or Clarithromycin (n =2).

### Prediction of amoxicillin resistance using SVM

**Figure 6:**
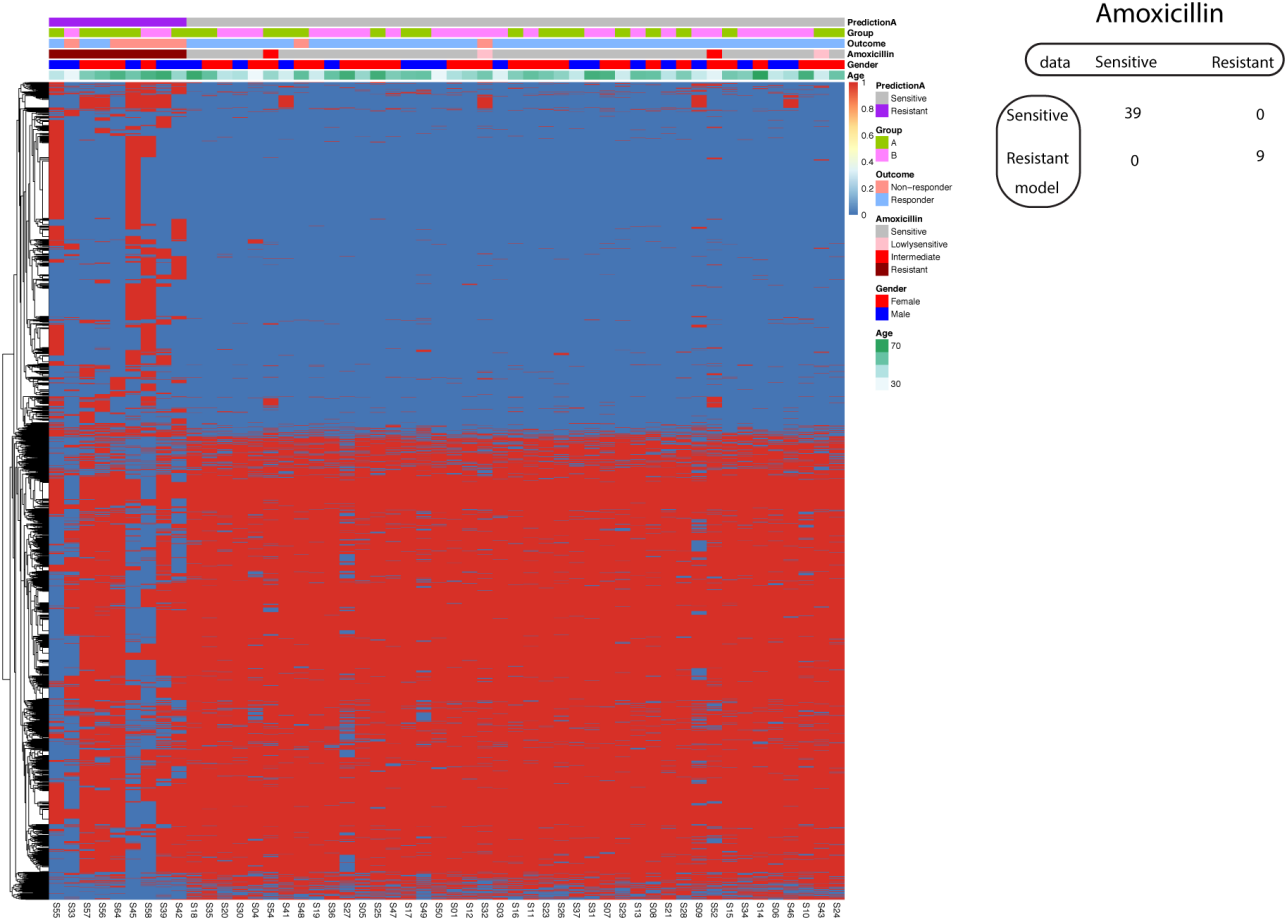
Support vector machine modeling of Amoxicillin resistance. SNP-patient matrix was displayed as a heatmap, with each row indicating one SNP site and each column one patient. Model Prediction was color coded as Sensitive (Grey) and Resistant (Purple); Group was color coded as gastric mucosa protection drugs A (Light Green) and B (Pink); Treatment outcome was color coded as non-responders (Orange) and Responders (Blue). Amoxicillin sensitivity is color coded as sensitive (Grey), lowly sensitive (Pink), intermediate (Red) and resistant (Dark red); Gender is color coded to indicate male (Blue) and female (Red); Age is color coded from white to Green. The confusion matrix for the entire cohort was also shown.

Using the known A1834G of 23S rRNA, the univariate model achieved 100% specificity and only 22.2% sensitivity. The mean sensitivity and specificity for single SNP model are only 0.45 and 0.46. All single SNP models with 100% specificity have sensitivity less than 50%. Therefore, we aimed to investigate the application of the supervised machine learning algorithm SVM in developing a predictive model for Amoxicillin resistance. Here we employed all significantly different SNP sites between resistant and sensitive strains to train SVM model. The feature selection procedure reduced the number of dimensions for the classification problem. Through this approach, we successfully generated an SVM model with 100% sensitivity and 100% specificity in predicting sensitive and resistant *Hp* strains (**Figure 6**). For the four strains (two lowly sensitive strains and two intermediate strains regarding Amoxicillin response) not used in model training, the model predicted all four strains as Amoxicillin sensitive.

Although this model allows us to obtain a good tradeoff between sensitivity and specificity, it is subjected to potential bias as we have much more variables than observations. To maximize the generalizability of the model, we implemented two validation procedures. In the first procedure, the original dataset was randomly split into training set (60%) and test set (40%). We obtained a model with 66.6% sensitivity and 100% specificity in the test set. In the second procedure, we employed 5-fold cross-validation. All five resulted models displayed 100% specificity, while the maximal sensitivity obtained is 50% in three models.

### Prediction of Clarithromycin resistance using SVM

**Figure 7:**
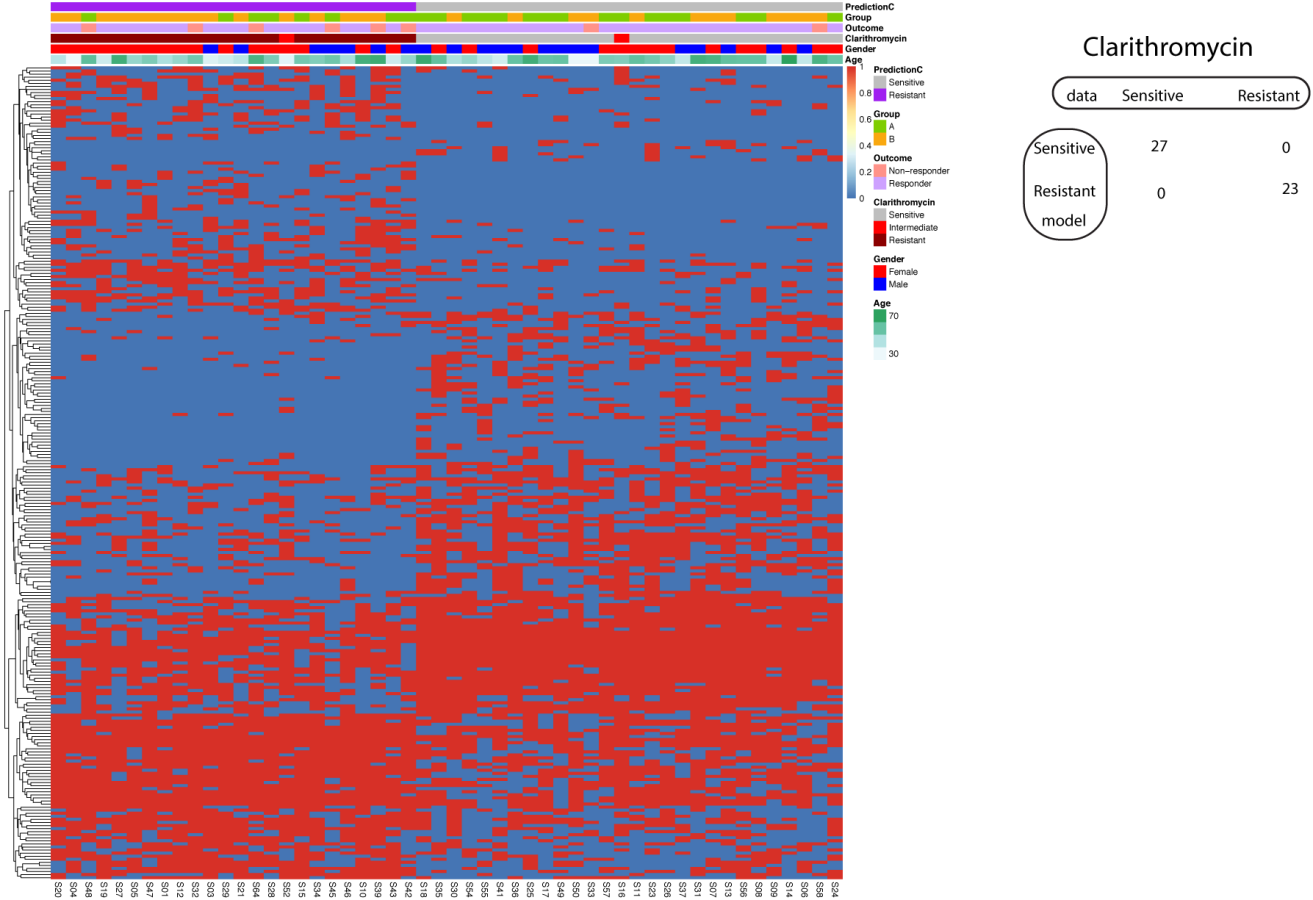
Support vector machine modeling of Clarithromycin resistance. SNP-patient matrix was displayed as a heatmap, with each row indicating one SNP site and each column one patient. Model Prediction was color coded as Sensitive (Grey) and Resistant (Purple); Group was color coded as gastric mucosa protection drugs A (Light Green) and B (Pink); Treatment outcome was color coded as non-responders (Orange) and Responders (Blue). Clarithromycin sensitivity is color coded as sensitive (Grey), intermediate (Red) and resistant (Dark red); Gender is color coded to indicate male (Blue) and female (Red); Age is color coded from white to Green. The confusion matrix for the entire cohort was also shown.

Using the known A2147G on the 23S rRNA gene, the model to predict Clarithromycin resistance demonstrated 87% sensitivity and 96% specificity. We further asked whether we could improve the model performance if we incorporate all significantly different SNP between Clarithromycin sensitive and resistant strains. Consequently, our investigation delved into the application of a supervised machine learning algorithm, specifically Support Vector Machine (SVM), in the development of a predictive model for Clarithromycin resistance. Remarkably, our efforts yielded a model that achieved 100% sensitivity and 100% specificity in predicting Clarithromycin-sensitive and resistant Hp strains (**Figure 7**).

Similar to the case of Amoxicillin resistance prediction with SVM, the resulted model strikes a perfect balance of sensitivity and specificity that is subjected to overfitting. The validation was implemented by two procedures. In the split procedure (60% training and 40% test), the model reached 100% sensitivity and 100% specificity in the test data. Using 5-fold cross-validation, all five resulted models exhibited 100% sensitivity and 100% specificity.

## Discussion

Whole genome sequencing had been successfully adopted in the clinic for Mycobacterium tuberculosis (Witney et al., 2016) and played important roles in identification of mutant strains of SARS-Cov-2 (Oude Munnink et al., 2020). In our study, we employed a comprehensive whole-genome sequencing approach to unravel the intricate antibiotic resistance patterns of *Hp*. Although antimicrobial resistance in *Hp* is a global threat to human health and the underlying molecular mechanisms have been explored in our study. In contrast to traditional phenotyping methods, which are characterized by their time-consuming nature and notable variability (Gerrits et al., 2006), our study showcases the remarkable advantages of employing Whole Genome Sequencing (WGS) for the prediction of *Hp* antibiotic resistance. The conventional approach, reliant on bacterial culture and drug testing, often consumes valuable time and yields results that are prone to experimental variation. In stark contrast, WGS offers an efficient, rapid, and precise alternative that holds immense potential in clinical setting.

Our research did not perform an exhaustive comparison between existing methodologies for predicting Helicobacter pylori (*Hp*) resistance and our proposed approach. The primary objective of our study was not to develop an optimal model, but rather to investigate the capabilities of machine learning in predicting Hp resistance. It is important to note that our final model might be subject to overfitting due to the limited size of our dataset, despite implementing cross-validation techniques. Traditional studies employing WGS typically rely on univariate statistical methods. One study frequently analyzed resistance-associated sites through kappa concordance analysis to identify both known and novel Hp resistance loci in WGS data (Zhou et al., 2022). Another study has concentrated on recognized drug resistance genes, like multidrug-resistant efflux pump genes (Iwamoto et al., 2014). The prediction of antimicrobial resistance based on WGS and machine learning has been explored using various supervised learning algorithms, particularly in studies involving the whole genome sequencing of Escherichia coli (Ren et al., 2022). Our study introduces a novel feature selection strategy for constructing machine learning models, diversifying the methodological approaches in this field.

Our findings underscored the challenge of achieving good sensitivity and specificity concurrently with models based on a single SNP site. Notably, a rare exception emerged with the single A2147G mutation on the 23S rRNA, which exhibited high sensitivity and specificity in predicting Clarithromycin resistance. This alignment with previous research corroborates the clear link between Clarithromycin resistance in *Hp* and A2146 and A2147 mutations (Lauener et al., 2019). We further demonstrated the efficacy of constructing phylogenetic trees based on significant SNP sites, revealing substantial separation between antibiotic-sensitive and resistant strains. These results suggest that future investigations aimed at building predictive models utilizing a combination of SNP sites could be a fruitful avenue to explore.

The complex nature of antimicrobial resistance in *Hp* underscores the limitations of predictive modeling based on single SNP sites (Reygaert, 2018). For instance, *Hp*’s resistance to Levofloxacin is likely linked to gyrase, an enzyme responsible for DNA negative supercoiling. Point mutations at amino acids 87, 88, 91, and 97 have been identified as potential Levofloxacin resistance determinants (Moore et al., 1995). Nonetheless, our study did not identify a single SNP site with the dual qualities of high sensitivity and specificity in predicting Levofloxacin resistance.

The challenging balance between high sensitivity and specificity for models based on single SNP sites primarily stems from the diverse mechanisms that underlie antibiotic resistance in *Hp*. Despite the promising potential of whole-genome sequencing in clinical medicine, our study does have several limitations. Firstly, our supervised machine learning approach relied on accurate phenotyping through traditional culturing and drug testing, which are susceptible to experimental variations (Su et al., 2019). Secondly, genome sequencing and drug testing of a single *Hp* colony oversimplify the complex clinical reality, where patients may harbor heterogeneous *Hp* populations within an intricate ecosystem of coexisting microbiota.

The construction of predictive modeling of antibiotic resistance was enhanced by feature selection. Significantly different SNP sites between sensitive and resistant strains were used as model input. In the case of Amoxicillin resistance, the best model has a sensitivity of 66% and a specificity of 100% in test data, while the known A1834G of 23S rRNA achieved 100% specificity and only 22.2% sensitivity. For Clarithromycin resistance, all trained models exhibited 100% sensitivity and 100% specificity in both training data and test data. This improved the performance of the known A2147G on the 23S rRNA gene, which has a sensitivity of 87% and a specificity of 96%. As proof-of-concept, predictive modeling using SVM with feature selection could lead to diagnostic model with higher predictive power.

Our study used sensitivity and specificity to evaluate the diagnostic models derived from different approaches. It’s important to note that sensitivity and specificity are inversely related. Increasing one often leads to a decrease in the other. The ideal model would have both high sensitivity and high specificity, but in practice, a balance is usually sought based on the consequences of false positives and false negatives. In a clinical setting, these metrics guide the choice of a diagnostic test based on what is more critical: not missing the condition (high sensitivity) or not incorrectly diagnosing it when it’s not there (high specificity). High sensitivity ensures resistant strains are correctly identified and treated.

One major limitation of our study is the small patient cohort. In the case of Amoxicillin drug resistance, we only have nine resistant strains. This limits our ability to train models with low variance. If the test dataset is small, we could have few or no resistant strains for Amoxicillin. In our small cohort, we did not have any resistant strain for Furazolidone and we only had one resistant strain for Tetracycline. This renders it impossible to address Furazolidone or Tetracycline resistance. Nevertheless, our dataset might be used for integration with other accessible datasets in the community for future predictive modeling efforts.

Taken together, the intricate antibiotic resistance patterns revealed by our whole-genome sequencing approach emphasize the need for a holistic perspective in understanding the dynamics of antimicrobial resistance in *Hp*. The lack of single SNP sites with simultaneous high sensitivity and specificity underscores the multifaceted nature of resistance mechanisms that this pathogen employs. As such, the incorporation of supervised machine learning techniques may hold the key to achieving more accurate and reliable predictive models for antibiotic resistance. Our findings emphasize the need for continued multidisciplinary research efforts that bridge genomics, microbiology, machine learning, and clinical medicine.

## Methods

### Sample collection and ethical approval

In total we collected sample from 52 patients with *Hp* infections. All enrolled patients have no intake of antibiotics within one month, no consumption of PPI or Chinese traditional medicine within two weeks. UBT test was employed to confirm the positivity of *Hp* infection. Biopsy samples were preserved in cultivation media and transported to laboratory at 4 degree. The studies involving human participants were reviewed and approved by the seventh Medical Center of PLA General Hospital Ethics Committee (No.2017-74) and other departments of gastroenterology from different hospitals applied and followed this content’s introduction. The patients/participants provided their written informed consent to participate in this study.

### Stratification by treatment outcome

Patients were divided into two groups (A and B). The patients in the group A were treated with 1000 mg tid of hydrotalcite, 20 mg bid of rabeprazole, 1000 mg bid of amoxicillin and 500 mg bid of clarithromycin; The patients in group B were treated with colloidal bismuth pectin 300 mg bid + rabeprazole 20 mg bid + amoxicillin 1000 mg bid + clarithromycin 500 mg bid for 10 days. At least 28 days after the end of treatment, all patients received ^13^C urea breath test to evaluate and compare the *Hp* eradication rate between two groups.

### *Hp* culture

Biopsy was inoculated onto Columbia blood agar plates supplemented with designated antibiotics. Inoculation was performed by direct contact of mucosa side and agar. The contact is gentle and even to ensure successful inoculation. Preservation media was applied to evenly cover the whole plate. The biopsy was removed and used in to confirm the presence of *Hp*. Agar plates were incubated in 5% O_2_, 10% CO_2_, 85% N_2_ at 37 degree for 3 or 4 days.

### Single colony inoculation and cryo-preservation

*Hp* colony was validated with morphology (Gram negative) and biochemical assays. Single *Hp* colony was transferred to a new Columbia agar plate and inoculated evenly. After 3-4 days of culture in incubator with 5% O_2_, 10% CO_2_, 85% N_2_ at 37 degree, *Hp* colonies were cryo-preserved in -80 degree for next generation sequencing.

### Antibiotic test

Determination of antibiotic sensitivity was performed with disk diffusion test according to standardized Kirby-Bauer procedure. White paper disks containing antibiotics were arranged onto the agar plates. The size of circular zones of poor bacteria growth surrounding paper disks was measured and used to define antibiotic sensitivity using customized thresholds (**Supplementary Figure 1**).

### DNA extraction

DNA extraction was performed with Magen HiPure Bacterial DNA kits. Final DNA output was dissolved in 30 μl TE buffer and analyzed with Qubit (Thermofisher) to determine concentration.

### Library construction and sequencing

10 ng DNA was used for library construction. DNA was fragmented with sonification to obtain fragments of 300 bp. Beads-based selection was performed to select DNA fragments after end repairing and adapter ligation. Library amplification was performed with Super Canace High Fidelity enzyme. Amplified libraries and size selected and purified. Agilent 2100 was used for library QC. The resulted libraries were normalized by molar concentration and sequenced with paired-end 150 bp mode on illumina platform.

### Mutation calling

Our mutation calling process commenced with rigorous quality control measures aimed at eliminating adapters and low-quality reads, ensuring the generation of clean reads. These clean reads were then meticulously aligned to the Helicobacter pylori reference genome, specifically the Helicobacter pylori 26695 genome (accessible at https://www.ncbi.nlm.nih.gov/nuccore/AE000511) using BWA (v0.7.17) with parameters “mem -t 2 -M -Y”. The resulting alignment data, in BAM format, was sorted and indexed with samtools (v1.9), which was the basis for mutation calling through the use of bcftools (v1.9) with parameters “call -vmO z”. To enhance the accuracy of our findings, only SNP sites demonstrating at least 2x coverage across all samples were retained for subsequent analysis. We performed “bcftools filter -O v -s LOWQUAL -e ‘\’’QUAL<10 || FMT/DP <5’\’’ –SnpGap 5 –set-GTs” to filter out SNPs of low quality. The resulting SNPs were annotated with Annovar (v191024).

### Identification of significant SNPs

The determination of significant SNPs was achieved via rigorous statistical assessments. Our analysis incorporated two independent statistical tests, namely the Chi-squared test and Fisher’s exact test. Employing a stringent approach, only SNPs exhibiting *p*-values below 0.05 in both tests were considered significant and subsequently utilized in our downstream analytical endeavors.

### Calculation of sensitivity and specificity

To gauge the efficacy of our approach for each antibiotic, we formulated confusion matrices specific to each significant SNP. The calculation of Sensitivity involved determining the ratio between true positives and model-predicted positives, while Specificity was calculated as the ratio between true negatives and model-predicted negatives. The confusion matrix is comprised of true positive (TP), false positive (FP), false negative (FN) and true negative (TN). To be precise, the formulars are “sensitivity = TP/(TP+FN)” and “specificity = TN/(TN+FP)”. These calculations were executed in the R programming environment.

### Phylogenetic analysis

Phylogenetic trees were constructed using SNP data identified in sample subsets of varying sizes, encompassing 5, 10, 20, 30, 40, and 50 samples, yielding 56671, 22601, 18749, 13938, 10397, and 3470 SNPs, respectively. Furthermore, we also plotted phylogenetic trees using only the significant SNPs identified through the aforementioned statistical tests, providing a comprehensive visual representation of genetic variation. The brief step for phylogenetic analysis is as follows. The SNPs in VCF file were converted to FASTA format and imported to MEGA (v10.2), then maximum likelihood tree was constructed with default parameters. The resulting tree file (nwk fomat) was then imported to an online tool called iTOL (https://itol.embl.de) for visualization.

### Support vector machine

To train classifier to predict antibiotic sensitivity using SNP information, input data was prepared using significant SNP for antibiotic under investigation. To facilitate the SVM modeling process, SNP data was systematically converted into numeric values, serving as the input for our SVM model. Briefly, input data was a patient-SNP matrix consisting of 0 or 1. SNP value of “1” suggests a polymorphism (non-wild type status), while SNP value of “0” indicates same genotype as the reference strain.

For Amoxicillin antibiotic resistance, we had 39 sensitive strains, 9 resistant strains, 2 intermediate strains and 2 lowly-sensitive strains. For Clarithromycin, we had 27 sensitive strains, 23 resistant strains and 2 intermediate strains. As the label “intermediate” and “lowly-sensitive” had very few observations, we aimed to build the two class SVM classifier using only sensitive and resistant strains. As for Clarithromycine, we had 27 sensitive strains, 23 resistant strains and 2 intermediate strains. Only sensitive and resistant strains were used for SVM.

The implementation of Support Vector Machine (SVM) modeling was facilitated through the e1017 package in the R programming environment. Employing a “radial” kernel, with a cost set at “1” for optimal SVM performance, we ensured model reproducibility by utilizing set.seed before the SVM modeling process. Sensitivity and specificity metrics were calculated using the above mentioned formulars: “sensitivity = TP/(TP+FN)” and “specificity = TN/(TN+FP)”.

In the model validation process for the Support Vector Machine (SVM) analysis, two distinct validation procedures were implemented to ensure the robustness and reliability of the model. The first procedure involved partitioning the original dataset into two subsets: 60% of the data was used as the training set, where the SVM model was trained to understand the patterns and relationships in the data. The remaining 40% constituted the test set, which was utilized to evaluate the model’s performance, specifically its ability to accurately predict outcomes on new, unseen data. This approach of splitting the dataset provides a straightforward way to check the model’s efficacy and generalizability.

The second procedure employed was 5-fold cross-validation, a more rigorous validation technique. In this method, the dataset was divided into five equal parts. In each of the five iterations of the process, a different fold was used as the validation set, while the remaining four folds collectively served as the training set. This cycle ensured that each part of the dataset was used both for training and validation. Cross-validation is particularly valuable as it mitigates the risk of overfitting, ensuring the model’s performance is not overly tailored to a specific subset of data. By averaging the results from all five folds, a more comprehensive and reliable assessment of the model’s performance is obtained, enhancing confidence in its predictive accuracy. Together, these two validation procedures provide a thorough examination of the SVM model’s capabilities, contributing to its credibility and applicability in practical scenarios.

## Data availability statement

All the raw Hp WGS data and SNP results were uploaded to the public repository zenodo (doi:10.5281/zenodo.10369064), can be accessed via the url: https://zenodo.org/records/10369064. Further inquiries can be directed to the corresponding authors.

## Ethics statement

The studies involving humans were approved by No.2017-74 ethical identification from the 7th Medical Center of PLA General Hospital. The studies were conducted in accordance with the local legislation and institutional requirements. Written informed consent for participation in this study was provided by the participants’ legal guardians/next of kin.

## Author contributions

JY: Data curation, Funding acquisition, Investigation, Project administration, Resources, Supervision, Writing – original draft, Formal Analysis. YJ: Data curation, Formal Analysis, Investigation, Project administration, Resources, Supervision, Writing – review & editing, Conceptualization. QY: Data curation, Methodology, Software, Validation, Visualization, Writing – original draft, Writing – review & editing. LL: Data curation, Investigation, Project administration, Resources, Writing – review & editing. CL: Conceptualization, Data curation, Investigation, Project administration, Resources, Writing – review & editing. BC: Methodology, Software, Supervision, Validation, Writing – review & editing. PZ: Data curation, Formal Analysis, Project administration, Writing – review & editing. XL: Conceptualization, Data curation, Formal Analysis, Investigation, Resources, Writing – review & editing. HL: Formal Analysis, Investigation, Methodology, Resources, Writing – review & editing. YS: Data curation, Formal Analysis, Resources, Validation, Writing – review & editing. XZ: Conceptualization, Data curation, Methodology, Writing – review & editing. YH: Data curation, Formal Analysis, Investigation, Methodology, Resources, Validation, Writing – review & editing. XH: Investigation, Methodology, Software, Supervision, Validation, Writing – original draft, Writing – review & editing. SL: Conceptualization, Data curation, Funding acquisition, Investigation, Resources, Writing – review & editing. YP: Conceptualization, Methodology, Project administration, Resources, Supervision, Visualization, Writing – original draft, Writing – review & editing.

## References

[B1] Camorlinga-Ponce M. Gomez-Delgado A. Aguilar-Zamora E. Torres R. C. Giono-Cerezo S. Escobar-Ogaz A.. (2020). Phenotypic and genotypic antibiotic resistance patterns in helicobacter pylori strains from ethnically diverse population in Mexico. Front. Cell Infect. Microbiol. 10, 539115. doi: 10.3389/fcimb.2020.539115 33643927 PMC7905308

[B2] Dutilh B. E. Backus L. Edwards R. A. Wels M. Bayjanov J. R. van Hijum S. A. (2013). Explaining microbial phenotypes on a genomic scale: GWAS for microbes. Brief Funct. Genomics 12 (4), 366–380. doi: 10.1093/bfgp/elt008 23625995 PMC3743258

[B3] Gerrits M. M. van Vliet A. H. Kuipers E. J. Kusters J. G. (2006). Helicobacter pylori and antimicrobial resistance: molecular mechanisms and clinical implications. Lancet Infect. Dis. 6 (11), 699–709. doi: 10.1016/S1473-3099(06)70627-2 17067919

[B4] Iwamoto A. Tanahashi T. Okada R. Yoshida Y. Kikuchi K. Keida Y.. (2014). Whole-genome sequencing of clarithromycin resistant Helicobacter pylori characterizes unidentified variants of multidrug resistant efflux pump genes. Gut Pathog. 6, 27. doi: 10.1186/1757-4749-6-27 24995043 PMC4079918

[B5] Lauener F. N. Imkamp F. Lehours P. Buissonniere A. Benejat L. Zbinden R.. (2019). Genetic determinants and prediction of antibiotic resistance phenotypes in helicobacter pylori. J. Clin. Med. 8 (1), 1–14. doi: 10.3390/jcm8010053 PMC6351930 30621024

[B6] Liu Z. Deng D. Lu H. Sun J. Lv L. Li S.. (2020). Evaluation of machine learning models for predicting antimicrobial resistance of actinobacillus pleuropneumoniae from whole genome sequences. Front. Microbiol. 11, 48. doi: 10.3389/fmicb.2020.00048 32117101 PMC7016212

[B7] Malla M. A. Dubey A. Kumar A. Yadav S. Hashem A. Abd Allah E. F. (2018). Exploring the human microbiome: the potential future role of next-generation sequencing in disease diagnosis and treatment. Front. Immunol. 9, 2868. doi: 10.3389/fimmu.2018.02868 30666248 PMC6330296

[B8] Moore R. A. Beckthold B. Wong S. Kureishi A. Bryan L. E. (1995). Nucleotide sequence of the gyrA gene and characterization of ciprofloxacin-resistant mutants of Helicobacter pylori. Antimicrob. Agents Chemother. 39 (1), 107–111. doi: 10.1128/AAC.39.1.107 7695290 PMC162494

[B9] Mousavizadeh L. Ghasemi S. (2021). Genotype and phenotype of COVID-19: Their roles in pathogenesis. J. Microbiol. Immunol. Infect. 54 (2), 159–163. doi: 10.1016/j.jmii.2020.03.022 32265180 PMC7138183

[B10] Oude Munnink B. B. Nieuwenhuijse D. F. Stein M. O'Toole A. Haverkate M. Mollers M.. (2020). Rapid SARS-CoV-2 whole-genome sequencing and analysis for informed public health decision-making in the Netherlands. Nat. Med. 26 (9), 1405–1410. doi: 10.1038/s41591-020-0997-y 32678356

[B11] Ren Y. Chakraborty T. Doijad S. Falgenhauer L. Falgenhauer J. Goesmann A.. (2022). Prediction of antimicrobial resistance based on whole-genome sequencing and machine learning. Bioinformatics 38 (2), 325–334. doi: 10.1093/bioinformatics/btab681 34613360 PMC8722762

[B12] Reygaert W. C. (2018). An overview of the antimicrobial resistance mechanisms of bacteria. AIMS Microbiol. 4 (3), 482–501. doi: 10.3934/microbiol.2018.3.482 31294229 PMC6604941

[B13] Salih B. A. (2009). Helicobacter pylori infection in developing countries: the burden for how long? Saudi J. Gastroenterol. 15 (3), 201–207. doi: 10.4103/1319-3767.54743 19636185 PMC2841423

[B14] Smith S. M. O'Morain C. McNamara D. (2014). Antimicrobial susceptibility testing for Helicobacter pylori in times of increasing antibiotic resistance. World J. Gastroenterol. 20 (29), 9912–9921. doi: 10.3748/wjg.v20.i29.9912 25110421 PMC4123372

[B15] Su M. Satola S. W. Read T. D. (2019). Genome-based prediction of bacterial antibiotic resistance. J. Clin. Microbiol. 57 (3), 1–15. doi: 10.1128/JCM.01405-18 PMC6425178 30381421

[B16] van Opijnen T. Camilli A. (2012). A fine scale phenotype-genotype virulence map of a bacterial pathogen. Genome Res. 22 (12), 2541–2551. doi: 10.1101/gr.137430.112 22826510 PMC3514683

[B17] Versalovic J. Shortridge D. Kibler K. Griffy M. V. Beyer J. Flamm R. K.. (1996). Mutations in 23S rRNA are associated with clarithromycin resistance in Helicobacter pylori. Antimicrob. Agents Chemother. 40 (2), 477–480. doi: 10.1128/AAC.40.2.477 8834903 PMC163139

[B18] Wang D. Guo Q. Yuan Y. Gong Y. (2019). The antibiotic resistance of Helicobacter pylori to five antibiotics and influencing factors in an area of China with a high risk of gastric cancer. BMC Microbiol. 19 (1), 152. doi: 10.1186/s12866-019-1517-4 31272365 PMC6611032

[B19] Witney A. A. Cosgrove C. A. Arnold A. Hinds J. Stoker N. G. Butcher P. D. (2016). Clinical use of whole genome sequencing for Mycobacterium tuberculosis. BMC Med. 14, 46. doi: 10.1186/s12916-016-0598-2 27004841 PMC4804576

[B20] Wroblewski L. E. Peek R. M. Jr. Wilson K. T. (2010). Helicobacter pylori and gastric cancer: factors that modulate disease risk. Clin. Microbiol. Rev. 23 (4), 713–739. doi: 10.1128/CMR.00011-10 20930071 PMC2952980

[B21] Zhou Y. Zhong Z. Hu S. Wang J. Deng Y. Li X.. (2022). A survey of helicobacter pylori antibiotic-resistant genotypes and strain lineages by whole-genome sequencing in China. Antimicrob. Agents Chemother. 66 (6), e0218821. doi: 10.1128/aac.02188-21 35652644 PMC9211431

