# Supplementary material for: Deciphering complex antibiotic resistance patterns in Helicobacter pylori through whole genome sequencing and machine learning
Source: Front Cell Infect Microbiol. 2024 Jan 4;13:1306368. doi: 10.3389/fcimb.2023.1306368 (PMC10878306; doi:10.3389/fcimb.2023.1306368)
Supplement: Supplementary file 2 [file Image_1.pdf]

A

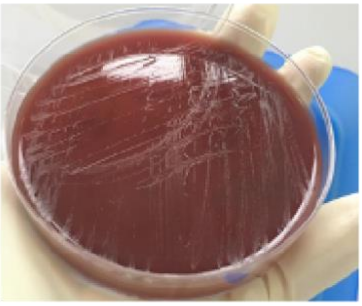

B

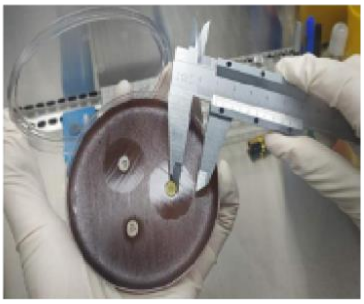

C

	Resistant	Intermediate	Sensitive
Clarithromycin	≤13	14-17	≥18
Tetracyclin	≤14	15-18	≥19
Levofloxacin	<13	13-17	>17
Furazolidone	≤14	15-16	≥17
Metronidazole	<16	16-21	>21
Amoxicillin	<14	14-17	>17

Supplementary Figure 1

(A) Columbia agar plate inoculated with one single colony of HP; (B) Disk diffusion test employed to determine antibiotic sensitivity; (C) Threshold of disk size used to determine antibiotic sensitivity.
